# Community service provider perceptions of implementing older adult fall prevention in Ontario, Canada: a qualitative study

**DOI:** 10.1186/s12877-018-0725-3

**Published:** 2018-02-01

**Authors:** Catherine S. Dykeman, Maureen F. Markle-Reid, Lorna J. Boratto, Chris Bowes, Hélène Gagné, Jennifer L. McGugan, Sarah Orr-Shaw

**Affiliations:** 1 Halton Region Health Department, 1151 Bronte Road, Oakville, ON L6M 3L1 Canada; 2 McMaster University School of Nursing, 1280 Main St. W., Health Sciences Centre, Room 3N25B, Hamilton, ON L8S 4K1 Canada; 3 Oxford County Public Health and Emergency Services, 410 Buller Street, Woodstock, ON N4S 4N2 Canada; 4 North Bay Parry Sound District Health Unit, 681 Commercial St, North Bay, ON P1B 4E7 Canada; 5 Ontario Neurotrauma Foundation, Suite 601, 90 Eglinton Ave E, Toronto, ON M4P 2Y3 Canada; 6 McMaster University School of Nursing, 1280 Main Street West, Hamilton, L8S 4L8 Canada; 7 Simcoe Muskoka District Health Unit, 15 Sperling Drive, Barrie, ON L4M 6K9 Canada

**Keywords:** Fall prevention, Implementation, Community, Services, Public health

## Abstract

**Background:**

Despite evidence for effective fall prevention interventions, measurable reductions in older adult (≥ 65 years) fall rates remain unrealized. This study aimed to describe the perceived barriers to and effective strategies for the implementation of evidence-based fall prevention practices within and across diverse community organizations.

This study is unique in that it included community service providers who are not generally thought to provide fall prevention services to older adults, such as retail business, community support, volunteer services, community foundations, recreation centres, and various emergency services.

**Methods:**

Interviews and focus groups were conducted with a purposive sampling of providers (*n* = 84) in varied roles within diverse community-based organizations across disparate geographical settings.

**Results:**

Community service providers experience significant multi-level barriers to fall prevention within and across organizations and settings. The overall challenge of serving dispersed populations in adverse environmental conditions was heightened in northern rural areas. Barriers across the system, within organizations and among providers themselves emerged along themes of Limited Coordination of Communication, Restrictive Organizational Mandates and Policies, Insufficient Resources, and Beliefs about Aging and Falls. Participants perceived that Educating Providers, Working Together, and Changing Policies and Legislation were strategies that have worked or would work well in implementing fall prevention. An unintentional observation was made that several participants in this extremely varied sample identified expanded roles in fall prevention for themselves during the interview process.

**Conclusions:**

Community service providers experience disabling contexts for implementing fall prevention on many levels: their specific geography, their service systems, their organizations and themselves. A systemic lack of fit between the older adult and fall prevention services limits access, making fall prevention inaccessible, unaccommodating, unavailable, unaffordable, and unacceptable. Educating Providers, Working Together, and Changing Policies and Legislation offers promise to create more enabling contexts for community stakeholders, including those who do not initially see their work as preventing falls.

## Background

Despite the accumulating evidence that most falls are predictable and their injuries preventable, unintentional falls and fall-related injury rates among adults aged 65 years and older in Canada continue to be all too common, negatively impacting health care resources [1] and older adults’ quality of life [2]. An estimated 20–30% of community-dwelling older adults (≥ 65 years) in Canada fall each year [3]. Rigorous evaluations have determined many interventions to be effective in reducing falls or fall risk. For example, comprehensive assessment and subsequent modification of relevant factors can reduce fall rates by 24% in those at risk for a fall [4]. Despite such strong evidence of the effectiveness of these strategies, the estimated rate of falls among older adults has remained unchanged for decades [5] and more recently the rate of fall-related injuries has risen significantly among senior women and in those 65–75 years old living in the community [6]. It has been suggested that many interventions remain unused or are not feasible to implement in practice [7, 8]. Furthermore, the need for prevention efforts beyond the health care system is increasingly recognized [9, 10].

Evidence reviews have also encompassed older adults’ perceptions of fall prevention activities [11], and determinants of their participation [12] as well as factors affecting care providers’ implementation of fall prevention strategies within health services for older adults [13]. Little is yet known about the barriers and facilitators to effective implementation of fall prevention practices experienced across a wider range of community organizations. There is a need to establish a knowledge base about perceived barriers and effective strategies to enable the adoption and use of fall prevention evidence within and among diverse community organizations as health is significantly determined by non-medical factors lying outside of the health sector [14]. Such information may be useful to local public health agencies in better engaging diverse community organizations and providers in evidence-based fall prevention strategies, in order to reduce fall-related injuries and improve quality of life for older adults living in the community.

This study builds on a quantitative survey of community services representatives regarding their use of fall prevention practices, attitudes toward implementation, knowledge and capacity for engagement, collaboration in fall prevention, and organizational readiness to implement available evidence [15]. Participants represented a range of perspectives within organizations regularly providing services to older people within each community, from front-line delivery (for example, personal support workers), to practice integration (for example, program managers), to administrative decision-making (for example, business owner). Participants also represented a range of perspectives across organizations regularly providing services to older people within each community, including both healthcare and non-healthcare organizations. Healthcare organizations included those providing clinical information and medical management to individuals. For example, paramedics can arrange fall risk or home safety assessments through Community Referral by Emergency Medical Services (CREMS) or upon transporting to the hospital following an emergency response. Non-healthcare organizations included those providing social and instrumental resources for community living. For example, community support services can offer transportation for fall prevention, such as to the optometrist for vision assessment, the drug store for pharmacist review of medications, or a local recreation centre for strength and balance training. The quantitative study found that while the majority (90%) of participants reported using at least one evidence-based fall prevention practice, only 21% believed that staff had the knowledge and skills sufficient for implementation and a meagre 10% perceived that available resources could support fall prevention activities. This qualitative study describes the perceived barriers to and effective strategies for the implementation of evidence-based fall prevention practices within and across diverse community organizations described during focus groups and interviews with these same participants, adding context to these quantitative findings.

## Methods

### Design

A qualitative descriptive research design was used to: (a) explore the barriers and facilitators to effective implementation of evidence-based fall prevention practices across diverse community services, (b) generate rich and detailed data that contribute to the understanding of factors influencing the provision of fall prevention within a community services context, and (c) include voices from within the actual community services [16].

Purposive sampling was used to include a wide range of information-rich data sources (participants and organizations) in our aim to identify cross-cutting barriers and strategies to implementing fall prevention [17].

### Sampling and settings

As the burden of falls varies disproportionately within the population by age [18], gender [19], culture [20], economics [21], and social status [22], population differences may affect service provider perceptions of barriers and facilitators to implementing fall prevention. Therefore, the catchment areas of three different Public Health Units in Ontario, Canada: North Bay Parry Sound District Health Unit, Simcoe Muskoka District Health Unit, and York Region Public Health Services provided our sampling frame. These Health Unit areas represented diversity by their Health Region Peer Group’s geographic and socio-demographic characteristic [23], including land area, population density, percentage of population age 65+, and proportions of visible minorities (see Table 1).

**Table 1:** Study site characteristics

Health Unit	Health Region Peer Group^a^	Land area (km^2^)	Population density (persons/km^2^) (Census, 2011)	Population age 65+ (% of total) (Census, 2011)	Visible minorities (% of population) (NHS, 2011)
North Bay Parry Sound District	Sparsely populated urban-rural mix	16,801	7.4	19%	1.7%
Simcoe Muskoka District	Moderately populated urban centres	8736	58	16%	4%
York Region	Highly populated urban centres	1762	586.0	12%	43%

^a^A peer group is comprised of health regions that are similar in prominent geographic characteristics and across 24 socio-demographic variables

Within each Health Unit area, the researchers worked with designated Health Unit staff to identify a variety of community organizations known to have regular contact with older adults (≥ 65 years), regardless of whether they were known to provide fall prevention activities (e.g. a grocery store) so as to not work just with the usual health partners but purposely include organizations outside of the medical domain in keeping with Public Health’s ecological, all-of-community approach to address environmental as well as personal determinants of falls. The designated Health Unit staff contacted potential participants from differing levels (i.e. directors, managers, supervisors, frontline) within these organizations, purposely to provide widely varying perspectives. All who expressed interest in the study were given an information letter inviting participation. We aimed for a sample size of 15–20 individuals per site, for more interviews than would be considered sufficient for understanding common perceptions and experiences among a group of relatively homogeneous individuals [24], yet manageable within the resources available.

Using a common interview guide for consistency in questioning across geography, organizations and participant roles, a total of nine focus groups (3 per site), supplemented by 30 semi-structured interviews to accommodate individuals unable to attend a scheduled focus group, were conducted with 84 participants from community organizations within the participating Health Unit areas (North Bay Parry Sound *n* = 25, Simcoe Muskoka *n* = 21, and York Region *n* = 38). Questions asked what kind of fall preventing activities their organization currently provided, what additional activities their organization should or could do, what factors made implementing difficult and alternatively easy, as well as how the organization would decide what to implement for preventing falls, and what steps would be taken to implement something new. Volunteers who participated in the study received a $25 gift card.

On average, focus groups and interviews lasted 1 hour. Managers/directors were interviewed separately from their staff to create a more comfortable environment for sharing. Attempts were made to form focus groups consisting of service providers from a variety of organizations, professional backgrounds, and with differing expertise in fall prevention, in order to facilitate broad discussion [25].

### Data collection

Data were collected through individual semi-structured interviews and focus groups co-conducted by two Research Coordinators. The development of the common interview guide was informed by other studies into the barriers to implementation of fall prevention evidence [26], and the team’s community-based fall prevention experience. Questions addressed topics such as fall prevention activities that were currently provided by their organization, the types of services, support, education and information that were needed to implement fall prevention activities, the adequacy of these services and supports for implementing fall prevention activities, the factors that facilitated and hindered implementation of fall prevention activities, the strategies that were used to address barriers to implementation of fall prevention activities, and future strategies that they recommended (interview guide available on request). Actual questions were adjusted and modified for clarity and interpretation during the course of data collection. The group and individual interviews were audio-recorded. Nonverbal communication was observed and recorded in field notes [27].

### Data analysis

Data from all group and individual interviews were transcribed verbatim, cleaned, and analyzed using NVivo 10 software. Data collection and data analysis occurred concurrently in an iterative fashion, as per the constant comparative method [28]. Emerging codes and concepts within each category were discussed as a team during formal meetings, and discrepancies were resolved by consensus. This iterative process allowed the research team to revise the interview guide, gather data, and refine emerging categories.

To assure the trustworthiness of our data, we drew on Patton’s suggestion that each researcher have the qualifications to carry out the study [29]. Our research and knowledge user (decision-making) team included skilled researchers with methodological expertise and expertise in fall prevention, and managers and providers who contributed their practical administrative and clinical experience. A number of triangulations were used for greater completeness of the findings in describing multiple realities across complex community settings. First, data triangulation was conducted using multiple data sources including service providers, and managers/directors. Second, methodological triangulation was conducted using data obtained by different collection methods, with individual interviews providing more in-depth detail and focus group interaction exploring commonalities and differences in broader overviews. Thirdly, investigator triangulation was conducted using the project team that represented multiple disciplines. After the coding of barriers and effective strategies uniquely experienced by each participant was completed, the data was organized according to the major categories identified. Next, subcategories were identified reflecting narrower topical areas within the major categories. Descriptive grids were then constructed summarizing pertinent content across each group. Project team members collaboratively identified and described cross-cutting themes encompassing the diversity of perspectives from the clusters of related responses. Responses were not quantified by participant demographics, years of organizational experience or organization type as our goal was to describe perceived barriers and strategies across a wide range of participant experience rather than specific to individual characteristics. As part of the audit trail, memos were kept to document ideas and to track decisions made throughout the analysis.

## Results

Participant characteristics captured separately in a quantitative survey show the diversity achieved in our sampling and are provided here for context (see Table 2).

**Table 2:** Settings and Participants

	Total (*n* = 84)	
	*n*	%
Health Unit Area		
North Bay / Parry Sound	24	29%
Simcoe Muskoka	21	25%
York Region	39	46%
Type of Community Served		
Urban	34	40%
Rural	3	4%
Both Urban and Rural	47	56%
Provider Occupation		
Health Care Professional	41	49%
Health Care Worker	10	12%
Administration	10	12%
Other	9	11%
Emergency Services Provider	4	5%
Recreation/Fitness Leader	4	5%
Social Services Worker	4	5%
Retailer	1	1%
Volunteer	1	1%
Role in Organization^a^		
Director	8	10%
Manager	17	20%
Supervisor	8	10%
Direct Service Provider	44	53%
Other	6	7%
Highest Level of Education Achieved^a^		
Graduate Degree/Post-secondary	21	25.3%
Bachelor’s Degree	32	38.6%
College Diploma/Certificate	26	31.3%
Technical/Trade School	2	2.4%
High School	2	2.4%

^a^Numbers do not add up to 84 as a result of missing scores(*n* = 1)

Data were collected from service providers, experienced (median 10 years) in a variety of roles (front-line, supervisory, and administration), in a broad range of community organizations, in the catchment areas of three Public Health Units in Ontario (see Table 2). Participants (*n* = 84) were mostly women (87%), ranging from 23 to 68 years of age (median age = 50.5 years). The majority were either college (34%) or university (64%) graduates. According to field notes, not all participants saw themselves as providing fall prevention. A surprising observation of this extremely varied sample was that, over the course of the focus group process, some participants newly identified opportunities for providing fall preventing interventions in their work, most notably those who initially did not believe they made any contribution at all. For example, a grocery store owner commented that he kept a clean store to attract customers but what he called risk management and liability was the same as fall prevention in the focus group, and a transit service suggested drivers could help prevent older adult falls by regularly providing a steadying hand for safe entry and exit from their vehicles.

### Barriers

While many community service providers commented that both the winter weather and geographic spread of Ontario’s landscape could make providing services of any kind difficult, barriers specific to implementing fall prevention within community services were described. Themes emerging within and across organizations and providers included: limited coordination of communication, restrictive organizational mandates and policies, insufficient resources, and beliefs about aging and falls. Exemplary quotes are identified by site (1, 2 or 3) followed by participant number.

#### Limited coordination of communication

Participants identified that haphazard communication was a barrier to implementing effective fall prevention. Individual attempts to communicate and better coordinate services were felt to be too time-consuming, with the evaluation of efforts impossible in the absence of client follow-up across services. Without coordinated information sharing, organizations appeared to be unaware of the similarities and differences in their roles and services, leading to both duplication and gaps in the system. As one participant noted, “*...assessments are being done over and over again... but nobody is actually doing the intervention.”* (ID214). Disjointed and piecemeal fall prevention efforts resulted from organizations working independently rather than collectively.

#### Restrictive Organizational Mandates & Policies

Funding formulas, organizational mandates and policies also were seen to restrict the type and location of services that could be provided. *“There are some barriers there. And I think it’s the mandates of the various other organizations and what is funded and what isn’t.”* (ID314)*.* Such restrictions not only affected healthcare organizations but other services as well*.* For example, fall prevention programs providing strength and balance training were ineligible for funded medical appointment transportation services. Participants also described services only being available during limited hours thus making it difficult for seniors to attend recommended fall prevention activities.

*“So, the taxis for example... or alternative transportation, they have very limited hours. You are kind of turfed out of the system if it’s just for recreational activities because it is for medical appointments and things like that. So, I think transportation’s a major barrier, actually, for the elderly.”* (ID310).

At times, restrictions were externally imposed outside of the organization. For some, legislation determined what service could be provided for the client. *“Our hands are tied because of the legislation; we have to take them to the Emerg.”* (ID216). For others, discipline-specific regulatory bodies determined whether scope of practice included a particular evidence-based intervention.

*“One thing that would make it a lot easier for myself as a PSW, is if I was allowed to do exercises with my clients without the stipulation of there having already been a physiotherapist or occupational therapist there...somehow adding that into the scope of what we do.”* (1FG1).

#### Insufficient resources

Most participants perceived that there were insufficient resources available to provide fall prevention activities to all of those that needed them. Participants reported that what is available is oversubscribed, as *“...everywhere you go in the system there’s a wait list...”* (ID133). Many said they would like to provide more services and reach more people, but their organizations lacked the financial and human resources.

Insufficient resources were not only seen to affect the availability of services, but also the quality of the service provided. Participants did not feel they had time to update their knowledge and ensure that the best fall prevention practices were being used. Consistency in staff education also depended on the sufficiency of resources. *“The challenge ...with training staff that work primarily in the community is reaching everybody…. working different days, different times.”* (ID206).

Insufficient resources in the system also meant out-of-pocket expenses for older people. Participants said that financial constraints prevented many older adults from attending fall prevention activities or making environmental modifications, despite the best of recommendations being made as in “*...we can [make] recommendations ... They just don’t have money to put in the necessary safety equipment that they need.”* (ID323).

#### Beliefs about aging and falls

Service provider beliefs, related to older age and the nature of falls, were at times a barrier to implementing fall prevention among older people. Some participants felt that fall prevention was no longer appropriate for clients as, “*when they’re frail and elderly, it’s kind of late*...” (ID101). In addition, some reported that using available resources for older people was not always seen as worthwhile, such as when *“referring on to more specialised assessment, [providers are] like “No, don’t bother....”*” (ID 220)*.* For others, falls were seen as unpredictable “*accidents*” as “...*Oh, yeah, well that happens*.” (ID101). Some also reported working with providers who accepted falls as inevitable, for example “*Well, you know, this person’s old. Of course they’re going to fall*” (ID 220). Both age- and fall-related misbeliefs were barriers to implementing fall prevention.

### Strategies

While participants did not distinguish strategies that had actually been effective from untested ideas, three themes emerged regarding strategies to promote implementation of fall prevention activities: educating providers, working together, and changing policies and legislation.

#### Educating providers

Participants stressed that all providers needed to be educated on effective fall prevention strategies and resources, with some indicating that it was important enough to be mandatory and part of basic training and staff orientation.

Effective education was seen to be readily available, interactive and ongoing. Participants suggested that frequent reminders were needed, *“routine little follow-ups, like a... little PowerPoint that pops up in the email”* (ID138). Performance feedback was also identified as key to *“let them know ... ‘that was great... we could have considered this [other alternative]”* (ID138) suggesting that dialogue was important to turning fall prevention education into practice.

Participants further indicated that fall prevention was not just minding rules and regulations but being passionate about the prevention activities, and championing the possibilities for healthier aging and independence rather than predicting worst-case scenarios. Promoting fall prevention more broadly as ‘everybody’s business’ was also suggested as a way of *“highlighting [fall prevention] to those in the community who may not be health care providers*” (FG2).

#### Working together

In participants’ eyes, fall prevention required a community-wide approach, where *“....all the stakeholders ... think together to address the issue”* (ID137). Stakeholders were seen to include government and providers, as well as older adults, informal caregivers, and the community at large. Crossing these organization boundaries was deemed necessary for mutual understanding that would lead to team work and fall prevention activities that “work”*.* The reason for this was simple: *“if you work in a silo, and don’t consider other people’s lenses, your work can ultimately be ineffective”* (ID113).

Participants suggested that inter-agency relationships needed to be built by working in partnership. Such partnerships were believed to be important because “*when we have those relationships, we share the information****”*** (ID215). Information sharing was seen to provide clarity on roles and responsibilities and enable more efficient use of resources. Participants identified potential challenges related to service integration and maintaining the level of commitment needed, but felt there were potential gains as *“we learn from each other... And if you didn’t work in partnership, you would never have that benefit.”* (ID217).

#### Changing policies and legislation

Participants indicated that there were no standards for fall prevention and predicted that change would only occur if there was a champion. Participants looked to governments to be a champion and provide the overall vision in developing and implementing standards for fall prevention. Having governments take the lead was seen as a way to make fall prevention more consistent and universal. Participants felt that providing fall prevention activities would be easier and more streamlined if consistent messages and approaches were used across the province and across professions. Standardization was especially important for province-wide and multidisciplinary organizations, as long as the flexibility to accommodate an individual client or community remained.

Government action in the form of legislation and policies was seen to provide powerful incentives, especially if accompanied by resources dedicated to sustaining fall prevention initiatives. Many participants agreed that fall prevention activities would be provided more effectively if collaboration was not optional, as *“Some people are very much wanting to hold on to their mandate...it has to be legislated that we have to work together”* (ID323)*.*

Participants also discussed the importance of organizational mandates and policies to integrating fall prevention activities into business as usual.

## Discussion

The objective of this study was to explore the perceived barriers and effective strategies to implementation of fall prevention activities. Views were gathered through group and individual interviews of people who work in a variety of roles in community service organizations across rural, mixed urban-rural and urban communities in Ontario.

This study was unique in that it included community services other than healthcare providers expected to provide fall prevention to older adults in Canada through discipline-specific best practice guidelines and accreditation processes. In addition to healthcare workers, social services workers, volunteers, emergency responders, retailers, and recreation and fitness leaders participated. Some participants in our study newly identified how they could prevent falls in their work following focus group discussions and while this observation arose outside of our research question, it may bear further exploration in future studies.

While barriers and facilitators seem strongly related to each other, and as such are often collapsed into the same ‘influencing factors’ discussion, a systematic review and synthesis of qualitative studies suggests that more complex and multifactorial consideration of fall prevention implementation is needed [30]. With this in mind, we separately explored first a cumulative effect of the multiple barriers and then of the multiple strategies. This wider perspective considered the interplay of the identified influencing factors for concepts relevant to understanding community-wide fall prevention implementation.

### Barriers

Multilevel barriers in the current service system were noted, suggesting that service providers were fully aware of the complexities involved in implementing fall prevention in the community. Beyond the weather and geographic challenges that affected all service provision, the barriers experienced in implementing fall prevention day-to-day included: Limited Coordination of Communication that bred duplication and gaps in service, and poor public awareness of service availability; Restrictive Organizational Mandates and Policies that impeded greater integration of fall prevention practices or mitigation of service gaps; Insufficient Resources that limited program availability, burdened clients with service costs and shortchanged providers offering evidence-based fall prevention programming; and Beliefs about Aging and Falls that discourage older adult fall prevention efforts.

Accessibility, accommodation, availability, affordability, and acceptability describe five dimensions of access that reflect the fit between the service and the served [31]. Despite nearly all offering at least one evidence-based fall prevention practice, these providers noted that their fall prevention activities could be physically inaccessible due to environmental conditions, but also unaccommodating (due to limited coordination of communication and restrictive organizational mandates and policies), unavailable (due to restrictive organizational mandates and policies and insufficient resources), unaffordable (due to insufficient resources), and unacceptable (due to beliefs about aging and falls). Evidence of access barriers therefore suggests that the current system of fall prevention services may not be designed to reflect the characteristics and expectations of both providers and older adults. A cumulative lack of fit that limits access to fall prevention offers an alternative explanation for the engagement challenges described in this population [32] beyond the perceived noncompliance of individuals [33].

Our findings confirm barriers identified individually elsewhere, including limited collaboration and communication among community care providers and agencies involved in implementing fall prevention, the absence of system-level fall prevention policies and financial reimbursement mechanisms, perceived knowledge deficits, and provider attitudes [34–37]. In addition, beliefs and behaviours at individual, organisational, and societal levels are suggested to pose some risk to the implementation of fall prevention [30]. Current systemic barriers may then contribute to a context that both increases the inequities of falls and disables otherwise effective fall prevention practices despite implementation with fidelity.

### Strategies

In addition to educating the community-dwelling older adults they serve, brainstormed ideas for making the implementation of fall prevention easier were not limited to addressing the barriers previously expressed and included: Educating Providers to increase fall prevention competencies within organizations; Working Together to communicate and coordinate efforts across the community as a whole; and Changing Policies and Legislation to prioritize and sustain funding for fall prevention efforts across the system.

Although it is unclear whether these strategies are examples of good practice or untested ideas, the cumulative range reflects an ecological approach to older adult fall prevention similar to that conceptualized by Cohen and Swift [38] in their Spectrum of Prevention framework for child and adolescent injury prevention. Integrating fall prevention education within services strengthens clients’ knowledge and skills and promotes community knowledge, while prioritizing ongoing provider education. Working together can foster coalitions and networks and affect organizational practices, while changing policies and legislation can be achieved through influence. Our real-world findings describe a systems approach to fall prevention, and suggest a vision for comprehensive strategies that promises mutual influence and greater synergy than would be possible by implementing any one strategy on its own, even as part of a phased initiative.

Identified strategies likewise cumulatively describe supportive conditions for implementing fall prevention from the perspective of Implementation Science’s drivers of change [39]. Educating providers is a key way to develop, improve and sustain competencies in preventing falls. Educating providers in fall prevention knowledge and skills also addresses the perceived lack of trained and competent staff reported previously. Working together can create and sustain hospitable environments for coordination and cooperation, easing resource challenges that many identified in implementing fall prevention activities alone. The absence of one organization support, specifically a system for using evaluation data to improve implementation, is notable and may warrant further exploration. Changing policies and legislation can improve the fiscal, political and regulatory environments in which administrative leaders guide and support staff to implement fall prevention. Indeed, workplaces supportive of quality improvement initiatives reported stronger confidence levels and intentions to implement fall prevention given the opportunity. Conducive environments are a tipping point for appropriately implemented evidence-based fall prevention efforts to produce impact of noteworthy significance [40].

### Strengths and limitations

Consistent with our study design, we intentionally gathered divergent views to provide the wide range of perspectives important to understanding the perceived barriers to and effective strategies for the implementation of evidence-based fall prevention practices within and across diverse community organizations. The qualitative method may limit the generalizability of the results beyond this unique sample and setting, however our findings align well with evidence-based prevention [38] and implementation frameworks [39] published by others, suggesting transferability to similar contexts. In addition, actual strategies that had enhanced fall prevention efforts were not distinguished from untested strategies that were only proposed, leaving unanswered questions. Further research is needed to further explore the factors affecting the effectiveness of community-based fall prevention initiatives as well as the community collaborations that support them.

## Conclusions

The effective implementation of proven interventions is necessary but not sufficient to prevent falling among older adults in the absence of enabling contexts. Despite the reportedly high use of evidence-based fall prevention practices, and systems-thinking to improve the effectiveness of implementation, community service providers experience significant barriers to fall prevention in multiple contexts: their geography, their service system, their organizations and among themselves. Barriers that limit access to fall prevention may even fuel the inequities within falls among older adults. Strategies to increase fall prevention competencies among providers, foster collaboration among services (including those outside the health sector), influence organizational policies and practices and advocate for changes in legislation offer promise for creating more enabling contexts. How might professionals in local public health agencies work within communities to better enable the effective implementation of proven fall interventions?

Public health professionals in Ontario are required to work together with community partners [41] and have been doing so for decades. The task now is to determine public health professional practices that will facilitate moving knowledge of effective fall prevention into action as intended, as well as support the collaboration needed to promote more enabling contexts -- not working just with the usual health partners, but including those in other community sectors who may not have yet considered their role in fall prevention.

## Abbreviations

CREMS: Community Referral by Emergency Medical Services
